# Resistance Exercise in a Hot Environment Alters Serum Markers in Untrained Males

**DOI:** 10.3389/fphys.2020.00597

**Published:** 2020-06-23

**Authors:** Arezoo Eskandari, Mohamad Fashi, Ayoub Saeidi, Daniel Boullosa, Ismail Laher, Abderraouf Ben Abderrahman, Gerorges Jabbour, Hassane Zouhal

**Affiliations:** ^1^Department of Exercise Physiology, Faculty of Physical Education and Sports Science, Tehran University, Tehran, Iran; ^2^Department of Exercise Physiology, Faculty of Physical Education and Health Sciences, Shahid Beheshti University, Tehran, Iran; ^3^Department of Physical Education, Damghan Branch, Islamic Azad University, Damghan, Iran; ^4^INISA, Federal University of Mato Grosso do Sul, Campo Grande, Brazil; ^5^Sport and Exercise Science, James Cook University, Townsville, Australia; ^6^Department of Anesthesiology, Pharmacology and Therapeutics, Faculty of Medicine, University of British Columbia, Vancouver, BC, Canada; ^7^ISSEP Ksar Said, University of Manouba, Tunis, Tunisia; ^8^University of Qatar, Doha, Qatar; ^9^Université de Rennes, M2S (Laboratoire Mouvement, Sport, Santé) – EA 1274, Rennes, France

**Keywords:** cytokines, heat, resistance exercise, hypertrophy, nterleukin 6, Interleukin 15, untrained young

## Abstract

**Purpose:** We examined the effects of moderate resistance exercise (RE) on serum cortisol, testosterone, extracellular heat shock protein (HSP70), and interleukin (IL)-6 and IL-15 concentrations in untrained males in a hot environment.

**Methods:** Ten untrained young males (26 ± 3 years; 75.8 ± 6 kg; 177.4 ± 5.3 cm) performed two series of full body RE [3 sets of 8 to 10 repetitions, 30–60 s recovery between series with 70% of one maximal repetition (1-RM), with a rest period of 1 to 3 min between exercises] carried out in a random order in both heated (∼35°C) and thermoneutral (22°C) conditions. Serum concentrations of testosterone, cortisol, HSP70, and IL-6 and IL-15 were measured before, at the end, and 1 h after RE sessions. Participants in both groups consumed 4 ml of water/kg body mass every 15 min.

**Results:** There were time-related changes in testosterone, HSP70, and IL-6 (*P* < 0.001), and cortisol and IL-15 (*P* < 0.05). Levels of cortisol, HSP70, and IL-6 increased immediately for RE at 35°C, and testosterone and IL-15 levels were decreased. Changes in serum testosterone, HSP70, cortisol, and IL-15 and IL-6 levels were reversed after 1 h. A significant time × condition interaction was observed for IL-15 and HSP70 (*P* < 0.001), cortisol and IL-6 (*P* < 0.05), but not for testosterone (*P* > 0.05).

**Conclusion:** RE in a heated environment may not be appropriate for achieving muscle adaptations due to acute changes of hormonal and inflammatory markers.

## Introduction

Healthy humans regulate resting body temperature near ∼37°C, but environmental perturbations can expand the range of temperature regulation (ranging between ∼35°C and ∼41°C) without adverse health consequences (Sonna et al., 2000; Byrne et al., 2006). Exercise in a hot environment affects physiological and immunological responses (Romeo et al., 2008; Cosio-Lima et al., 2011; Suzuki, 2018), elevates heart rate responses, and imposes a higher physiological workload (Özgünen et al., 2010; Neal et al., 2016).

Exposure to heat stress modulates circulating levels of catecholamines (Febbraio, 2001), testosterone, cortisol (Vingren et al., 2016), and extracellular heat shock protein (HSP) (Benini et al., 2015). Exercise under heated environmental conditions can lead to dehydration due to changes in plasma volume caused by sweating and transient fluid movement into and out of the intravascular space (Zouhal et al., 2009). This reduction in plasma volume lowers blood supply to working muscles. Moreover, variations in plasma volume can potentially hamper the interpretation of blood biomarkers (Kargotich et al., 1998), which regulate inflammation and acute immune responses (Thomson and Lotze, 2003). Increases in stress and body temperature alter cytokine levels, muscle contractility and force transmission, metabolic rate, substrate partitioning, and anabolic status during exercise (Ferguson et al., 2002; Steinacker et al., 2004; Girard et al., 2015; Vingren et al., 2016; Suzuki, 2019). Increases in anabolic hormone concentrations after an acute bout of resistance exercise (RE) in the trained state promote muscle protein synthesis (Kraemer and Ratamess, 2005).

Although several studies examined the effects of endurance exercise in hot environments (Shephard, 1998; Sawka et al., 2015; Junge et al., 2016), there is little information on the acute responses to RE under conditions of heat stress. Heat stress stimulates mammalian target of rapamycin (mTOR) signaling after RE in physically active subjects (Kakigi et al., 2011) and also increases growth hormone (GH) release in females (by 85%) and males (by 107%) (Casadio et al., 2017). Highly trained male athletes undergoing RE in a heated environment (30°C and 40–60% relative humidity) experienced increases in both upper and lower body power production (Casadio et al., 2017), while lower body RE in the heat led to small improvements in lower and upper body strength in professional rugby athletes (Miles et al., 2019).

Collectively, these studies suggest enhanced anabolic responses in physically active and trained individuals performing RE in a hot environment. However, it is unclear if performing RE in the heat enhances the anabolic and performance responses in untrained individuals, because RE combined with heat exposure could induce an excessive stress response in untrained individuals when compared to trained individuals. We investigated the effects of a single session of moderate RE in the heat (35°C) on serum levels of cortisol, testosterone, HSP, IL-6, and IL-15 in untrained adult males. We tested the hypothesis that changes in IL-6, IL-15, testosterone, cortisol, HSP levels, and plasma volume in response to an acute RE session would be enhanced in the heat and could potentially improve anabolic responses similar to those occurring in physically active and trained individuals.

## Materials and Methods

### Participants

Ten healthy, untrained adult males (age: 26 ± 3 years; height: 177.4 ± 5.3 cm; body weight: 75.8 ± 6 kg, BMI = 24.2 ± 2.1) without cardiovascular or musculoskeletal diseases participated in this study. All participants were informed about the study design, risks, and possible benefits associated with the procedures and provided written informed consent before participation. Participants completed a physical activity questionnaire indicating their weekly physical activity levels before the study (Craig et al., 2003). Participants who performed 2 ± 1 h or less of recreational sports per week, but had no prior experience with RE, were selected for this study. Participants were advised to eat the same predetermined meals during the 2 days before blood sampling [total energy (kcal/day) = 2162 ± 150; total protein (g/day) = 81.08 ± 12; protein (g/kg BW/day) = 1.2; total protein (% energy) = 15; total carbohydrate (g/day) = 297.28 ± 16; total carbohydrate (% energy) = 55; total fat (g/day) = 72.06 ± 21; total fat (% energy) = 30]. The amount of nutrients consumed by the participants was calculated using a previously described method (McCance and Widdowson, 2014). Ethical approval was obtained from the Ethics Committee of the Tarbiat Modares University of Tehran, Iran (IR.Rec.1396005).

### Resistance Exercise Protocols

Participants engaged in two familiarization sessions of RE (separated by 48 h) a week before starting the study for load determination in each exercise. Thereafter, participants completed full body RE sessions on 2 days within the same week in random order (with a rest period of 72 h between them); these were done at the same time each day to avoid circadian influences. The two RE protocols used were as follows: RE under heated conditions (∼35°C, relative humidity 40%) and RE in thermoneutral conditions (∼22°C, relative humidity 40%). The RE protocol consisted of three sets each of 8–10 repetitions of bench presses, leg presses, lat pull-downs, leg extensions, shoulder presses, leg curls, biceps curls, and calf raises, with 70% of 1RM, 1 min of recovery between sets, and 3 min between the various exercises. Every repetition was performed at a moderately slow tempo (2-s concentric and 2-s eccentric contraction, with a 1-s pause between each) to maximize time under muscle tension. A 5-min warm-up was performed on a cycle ergometer with 60–70 rpm at 150 W before the RE sessions (Fröhlich et al., 2014).

Values for 1-RM were calculated using an indirect method before the first training session using a 10-RM test. The 1-RM equation used was Weight/[1.0278 – (0.0278 × maximum number of repetitions)] (Brzycki, 1993). If participants completed more than 10 repetitions, they repeated the test using heavier weights until they were unable to complete 10 repetitions (Brzycki, 1993; Mayhew et al., 1995; Banyard et al., 2017). Participants completed all sessions under the supervision of an experienced coach. The muscle loads experienced during both conditions were identical since the number of repetitions and sets and the intensity were constant for the two RE sessions (Baechle and Earle, 2008). The average error for 1-RM calculated by the Brzycki equation and predictive 1-RM was 6.80 for bench press, 4.53 for leg press, 5.89 for lat pull-down, 0.61 for leg extension, 6.35 for shoulder press, 5.44 for leg curl, 6.74 for biceps curl, and 0.61 for calf raises.

### Procedures in Heated and Thermoneutral Environments

**FIGURE 1:**
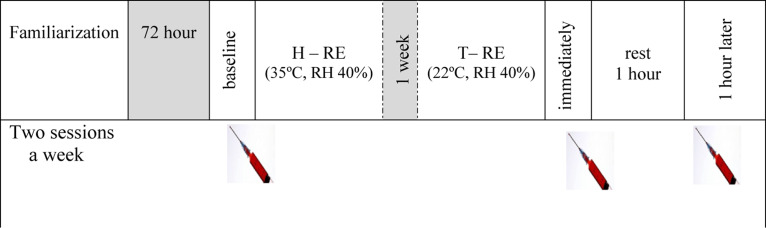
Schematic representation of experimental protocol. H-RE: heated resistance exercise, T-RE: thermoneutral resistance exercise.

Participants rested undisturbed in either a heated or thermoneutral environment for 30 min before the exercise sessions. RE in a heated environment started once the participant’s core temperature reached ∼35°C (which required about 30 min). The temperature and relative humidity of the environment were monitored using a Hot-Wire Anemometer (aTES-1341, Taiwan) and core body temperature was measured with a rectal thermistor (Data Therm II; RG Medical Diagnostics, Wixom, MI) placed ∼10 cm beyond the anal sphincter. Measurements were taken at 5-min intervals until the core temperature reached ∼35°C (Figure 1).

### Blood Samples

Blood samples (10 ml) were obtained at three time points: 1 h before the start of the protocols, immediately after RE sessions, and 1 h after RE sessions. Participants remained seated under thermoneutral conditions (22°C and 40% relative humidity) during these procedures. Half the blood sample (5 ml) was centrifuged (1,500 g, 4°C, 15 min), and the serum was stored at −80°C for later measurements of cortisol, testosterone, IL-6, IL-15, and extracellular heat shock protein-70 (HSP) concentrations using enzyme-linked immunosorbent assays. The assay kits were as follows: human cortisol (sensitivity: 2.44 ng/ml), human testosterone (sensitivity: 8 pg/ml), human IL-6 (sensitivity: 8 pg/ml), human IL-15 (sensitivity: 1.1 pg/ml), and human HSP-70 (sensitivity: 2.5 U/ml), all obtained from the same manufacturer (R&D Systems, United Kingdom). Intra- and inter-assay coefficients of variation were all <10% (Waller et al., 2017).

The remaining 5 ml of each blood sample was placed in a vacutainer tube with EDTA for determination of hematocrit and hemoglobin levels for the estimation of plasma volume variations (Dill and Costill, 1974; Rhibi et al., 2019). All measurements were made in duplicate by the same researcher to minimize inter-assay variations.

$$PVV\left(\%\right)=100\times \left\{\frac{H\,b\,1}{H\,b\,2}\times \frac{\left(1-H\,t\,2\times x\,10^{-2}\right)}{\left(1-H\,t\,1\times x\,10^{-2}\right)}\right\}-100$$

$$PVV\left(\%\right)=100\times \left\{\frac{H\,b\,1}{H\,b\,3}\times \frac{\left(1-H\,t\,3\times x\,10^{-2}\right)}{\left(1-H\,t\,1\times x\,10^{-2}\right)}\right\}-100$$

PVV(%): Percent plasma volume variations; 1: value measured at baseline; 2: value measured immediately after the protocol; 3: value measured 1 h post-exercise; Ht: hematocrit in %; Hb: hemoglobin in g/dl.

Serum markers were adjusted for plasma volume changes using the following formula (Sherk et al., 2013):

Corrected value = Uncorrected value × [(100 + %?PV)/100].

### Statistical Analysis

A power analysis (power = 0.80, and alpha error = 0.05) indicated that a sample size of *n* = 10 per group was needed to identify a statistically significant group by time interaction effect based on previous studies (Stadnyk et al., 2018). A Shapiro–Wilk test was used to confirm a normal distribution of variables. Data on the condition (heat vs thermoneutral) or time (1 h pre-protocol, immediately after protocol, or 1 h post-protocol) were analyzed using a repeated-measures ANOVA. Effect sizes (ES) were determined from ANOVA output by converting partial eta-squared values to Cohen’s d values. Within-group ES were computed using the following equation: ES = (mean post – mean pre)/SD (Cohen, 1988). ES were considered trivial (<0.2), small (0.2–0.6), moderate (0.6–1.2), large (1.2–2.0), and very large (2.0–4.0) (Batterham and Hopkins, 2006). In cases of a significant time effect, an ANOVA with Fisher’s least significant differences *post hoc* test was performed to determine differences between groups. Results are expressed as mean ± SD with levels of significance set at *p* < 0.05.

## Results

Plasma markers were measured before, immediately at the end, and 1 h after RE in participants from the two exercise groups (in heated vs thermoneutral conditions) and are shown in Table 1. Shapiro–Wilk tests indicated a normal distribution of cortisol (*p* = 0.132), testosterone (*p* = 0.761), HSP70 (*p* = 0.711), IL-6 (*p* = 0.238), and IL-15 concentrations (*p* = 0.315) in thermoneutral conditions, and for cortisol (*p* = 0.421), testosterone (*p* = 0.345), HSP70 (*p* = 0.125), IL-6 (*p* = 0.241), and IL-15 concentrations (*p* = 0.433) in heated conditions.

**TABLE 1:** Mean ± SD, serum concentrations of CO (cortisol), TC (testosterone), IL-6, HSP70, IL-15 and PVV (plasma volume varation) in the baseline (1-h pre-protocol), immediately after protocols, and 1-h later in the H-RE (heated resistance exercise) and T-RE (thermoneutral resistance exercise) conditions.

Variables	Baseline		Immediately			1 h later		
	T-RE	H-RE	T-RE	H-RE	*P*	T-RE	H-RE	*P*
CO (ng/ml)	132.01 ± 14.3	132.64 ± 12.8	158.58 ± 12.5	178.27 ± 10.6	0.001*	139.49 ± 22.9	134.18 ± 19.9	0.058
	CVa = 0.10	CVa = 0.09	CVa = 0.07	CV = 0.05		CVa = 0.16	CVa = 0.14	
	CVb = 0		CVb = 12%			CVb = −4%		
TC (ng/ml)	2.70 ± 0.67	2.70 ± 0.77	5.10 ± 1.06	3.90 ± 0.95	0.031*	4.30 ± 0.98	3.30 ± 0.79	0.34
	CVa = 0.24	CVa = 0.28	CVa = 0.20	CVa = 0.24		CVa = 0.22	CVa = 0.23	
	CVb = 0		CVb = −24%			CVb = −23%		
IL-6 (Pg/ml)	6.02 ± 0.54	6.02 ± 0.17	9.17 ± 0.43	8.57 ± 0.12	0.004*	7.69 ± 0.76	8.31 ± 0.17	0.012*
	CVa = 0.08	CVa = 0.02	CVa = 0.04	CVa = 10		CVa = 0.09	CVa = 0.02	
	CVb = 0		CVb = 7%			CVb = −7%		
HSP70 (Pg/ml)	0.33 ± 0.12	0.35 ± 0.17	0.95 ± 0.07	1.17 ± 0.12	0.021*	0.48 ± 0.16	0.31 ± 0.17	0.22
	CVa = 0.36	CVa = 0.48	CVa = 07	CV = 10		CVa = 0.33	CVa = 0.54	
	CVb = 0		CVb = 24%			CVb = −35%		
IL_15 (Pg/ml)	95.6 ± 22.9	100.4 ± 16.7	362.3 ± 29.7	340.0 ± 29.9	0.043*	321.0 ± 24.4	291.6 ± 26.7	0.013*
	CVa = 0.23	CVa = 0.16	CVa = 0.08	CVa = 0.08		CVa = 0.07	CVa = 0.09	
	CVb = 0		CVb = −6%			CVb = −9%		
PVV (%)	0	−0.8	−6.4 ± 1.3	−7.7 ± 1.9	0.102	−2.6 ± 2.3	−3.1 ± 2.2	0.301
			CVa = −0.20	CVa = −0.24		CVa = −0.88	CVa = −0.70	
			CVb = −20%			CVb = −19%		

CVa = Intra-assay coefficient of variation. CVb = Inter-assay coefficient of variation. *Significantly different among groups (*P* ≤ 0.05).

There were no differences at baseline for serum markers for the participants in either of the two exercise groups. Significant time-related effects were detected for cortisol (*p* = 0.030, ES = 0.963), testosterone (*p* = 0.001, ES = 0.699), HSP70 (*p* = 0.001, ES = 0.866), IL-6 (*p* = 0.001, ES = 0.876), and IL-15 concentrations (*p* = 0.042, ES = 0.844). Levels of cortisol (*p* = 0.001; ES = 0.965), IL-6 (*p* = 0.004; ES = 0.832), and HSP70 (*p* = 0.021; ES = 0.651) were higher immediately after RE under heated conditions. There were reductions in testosterone (*p* = 0.031; ES = 0.753) and IL-15 (*p* = 0.043; ES = 0.588) levels immediately after RE under heated conditions. Serum markers were lower 1 h after RE under heated conditions compared to the thermoneutral conditions (Table 1).

Plasma volume variation (%) values were lower following RE in a heated environment when measured immediately after exercise (*p* = 0.102; ES = 0.231) and also 1 h after exercise (*p* = 0.001; ES = 0.301).

A significant time × condition interaction was observed for IL-15 (*p* = 0.034, ES = 0.197), HSP70 (*p* = 0.001, ES = 0.76), cortisol (*p* = 0.004, ES = 0.336), and IL-6 (*p* = 0.003, ES = 0.956), but not for testosterone (*p* = 0.133; ES = 0.223).

## Discussion

This is the first study to examine acute plasma volume variations (an indicator of dehydration) and changes in serum levels of cortisol, testosterone, IL-6, HSP70, and IL-15 in untrained males following RE in a heated environment. The key findings of our study were that contrary to our hypothesis, serum concentration of IL-15 decreased, whereas cortisol, HSP70, and IL-6 levels increased immediately after RE in heated conditions. The concentrations of cortisol, HSP70, IL-6, and IL-15 returned to pre-exercise values 1 h after the RE session in both exercise groups. The changes in serum markers were associated with reductions in the plasma volume. These results suggest that resistance exercise in a heated environment may not mimic the anabolic responses measured under thermoneutral conditions.

Cortisol is a catabolic hormone released after exercise and exposure to heat (Brenner et al., 1997). Levels of cortisol increase in individuals who exercise in the heat (Brenner et al., 1998). Increases in plasma cortisol levels occur when body core temperature increases by at least 1.2°C (Brenner et al., 1998; Niess et al., 2003), and have also been measured in subjects exercising at 40°C (Brenner et al., 1997). Increased cortisol concentrations after RE are associated with muscle hypertrophy and power development (Smilios et al., 2003; Martorelli et al., 2020). Thus, our results of increases in cortisol levels immediately after RE, and 1 h after the end of RE, in a heated environment are in agreement with previous reports.

Concentrations of testosterone increase following different protocols of RE (Gotshalk et al., 1997; Bosco et al., 2000; Ratamess et al., 2004). A bout of moderate-intensity RE (70% 1 RM) increased testosterone concentrations (Rietjens et al., 2015), while repeated maximal sprints (2 sets of 5 × 15 s sprints) in the heat (35°C) did not change testosterone (Hoffman et al., 1996). Moreover, RE in a heated environment did not alter testosterone or cortisol concentrations (Casadio et al., 2017). Our findings of decreases in testosterone immediately, and 1 h after RE, in heated conditions may represent an indicator of hormonal variations to heat adaptations during RE (Beaven et al., 2008), and thermoregulatory responses to heat stress (Racinais et al., 2012). The different population groups and heat conditions we used (healthy, untrained males; 35°C) and those used by Casadio et al. (2017) (highly trained power athletes, 30°C) could account for the different outcomes of the two studies. Further studies are needed to determine the influence of these factors on the acute changes in testosterone levels after RE in the heat.

IL-6 is a myokine released by skeletal muscle in response to exercise (Walsh et al., 2011; Pedersen and Febbraio, 2012), but the association between IL-6 and muscle hypertrophy is not well studied (Serrano et al., 2008). IL-6 is an important regulator of satellite-cell mediated hypertrophic muscle growth (Serrano et al., 2008) and may have a role in muscle tissue remodeling, especially in response to muscle damage (Paulsen et al., 2012; Pedersen and Febbraio, 2012). Increases in IL-6 that occur immediately after RT could be present following 1 h of recovery, with values similar to those found in the current study (Nieman et al., 2004). Our study shows that serum IL-6 levels were higher immediately after exercise in both conditions (22°C and 35°C) and after 1 h of exercise only in the heated environment. A possible explanation for the surge in IL-6 during RT in heated conditions may be related to the intensity of the exercise (Cullen et al., 2016, #839). In the present study, heat stress may have been associated with increased exercise intensity, and as a result, it raises the level of IL-6 after 1 h of heat resistance exercise.

Exercise-induced cytokine release (e.g., IL-6) could also be associated with alterations in extracellular HSP levels that can stimulate anabolic responses (Noble et al., 2008; Pedersen, 2011). Most HSPs are intracellular proteins that bind to nascent peptides and proteins that facilitate proper assembly and folding (Benini et al., 2015). Increases in concentrations of HSPs protect against protein denaturation and cell death caused by adverse environmental conditions including heat stress (Calderwood et al., 2007; Noble et al., 2008). We report that HSP70 levels increased immediately after and decreased 1 h after RT under heated conditions. The increases in HSP70 levels we detected immediately after RT in a heated environment suggest that heat stress persists for at least 1 h after RT in heated conditions.

Another important finding of this study is the smaller increase in IL-15 immediately after RE, and 1 h after RE in heated conditions when compared to control conditions. IL-15 has anabolic and anti-atrophy properties and is increased by a single session of RE in both untrained and trained individuals (Riechman et al., 2004). Signaling pathways for IL-15/IL-15Rα are activated in skeletal muscle in response to a single session of RE (Perez-Lopez et al., 2018). While our data are in partial agreement with these findings, it is also possible that muscle damage due to the cumulative effects of heat stress and RE impairs the recovery process (Krentz and Farthing, 2010), since IL-15 acts directly on differentiated myotubes to increase muscle protein synthesis and reduce protein degradation (Quinn, 2008).

Our study indicates that RE in a heated environment could, at least partially, be influenced by muscle damage and induced increases in IL-6, cortisol, and HSP70, and decreases in testosterone and IL-15 levels. These changes were observed with minimal changes in plasma volume after training in heated and normothermic conditions. Exercise-induced dehydration in heated conditions decreases plasma volume and can affect the concentrations of circulating biomarkers (Kargotich et al., 1998). Increases in IL-6 and cortisol levels after exercise in the heat are related to the rate of fluid loss (Costello et al., 2018). The participants in our study consumed water (4 ml/kg of body mass every 15 min) to minimize changes in plasma volume under both conditions of exercise (22°C and 35°C).

### Study Limitations

We did not measure possible muscle damage caused by exercise in heated conditions. Another limitation of our study is that we measured changes in serum markers after a relatively short period (∼1 h) of RE, which may be insufficient to detect greater differences under these two conditions (22°C and 35°C) of exercise.

## Conclusion

Exercise in a heated environment impairs muscle adaptations and hypertrophy as measured by changes in serum hormonal and inflammatory biomarkers. More studies are needed on the long-term adaptations of different amounts and intensities of RE in a heated environment and other extreme environmental conditions.

## Data Availability Statement

The datasets generated for this study are available on request to the corresponding author.

## Ethics Statement

The studies involving human participants were reviewed and approved by Tarbiat Modares University of Tehran, Iran. The patients/participants provided their written informed consent to participate in this study. Ethical approval was obtained from the Ethics Committee of the Tarbiat Modares University of Tehran, Iran (IR.Rec.1396005).

## Author Contributions

AE, MF, AS, and HZ conceived and designed the research. AE, MF, and AS conducted the experiment. AE, MF, AS, and AB analyzed the data. AE, MF, AS, DB, IL, AB, GJ, and HZ wrote the manuscript. All authors read and approved the final version of the manuscript.

## Conflict of Interest

The authors declare that the research was conducted in the absence of any commercial or financial relationships that could be construed as a potential conflict of interest.

## Abbreviations

1-RM: 1 repeated maximum
CV: Coefficient of variation
ES: Effect size
F-test: Variation between sample means/variation within the samples
HSP70: 70 kilodalton heat shock protein
IL-15: Interleukin 15
IL-6: Interleukin 6
mTOR: Mammalian target of rapamycin
RE: Resistance exercise
RH: Relative humidity.
